# Supplementary material for: Quantifying upper limb motor impairment in people with Parkinson’s disease: a physiological profiling approach
Source: PeerJ. 2021 Feb 5;9:e10735. doi: 10.7717/peerj.10735 (PMC7869669; doi:10.7717/peerj.10735)
Supplement: Supplemental Information 2 [file peerj-09-10735-s002.docx]

The raw data provides each score for each trial of each test for each participant in the study. Parkinson's disease participants' scores provided during their 'off' medication state are coded '0;' Parkinson's disease participants' scores provided during their 'on' medication state are coded '1;' scores for all age- and sex-matched control participants are coded '2.'
